# A multilingual analysis of pro Russian misinformation on Twitter during the Russian invasion of Ukraine

**DOI:** 10.1038/s41598-024-60653-y

**Published:** 2024-05-02

**Authors:** Cameron Lai, Fujio Toriumi, Mitsuo Yoshida

**Affiliations:** 1 Graduate School of Engineering, The University of Tokyo, Tokyo, 113-0033 Japan; 2 Institute of Business Sciences, University of Tsukuba, Tokyo, 112-0012 Japan

**Keywords:** Misinformation, Networks, Text embedding, Twitter, Social media, Computational science, Information technology

## Abstract

The Russian government has long since engaged in an information campaign of propaganda and disinformation as a major part of foreign policy. This has been taken to new heights since the invasion of Ukraine in February 2022. In this study, we investigate pro-Russian misinformation within the opening weeks of the invasion in 6 languages: English, Japanese, Spanish, French, German, and Korean. Using Twitter data, we apply a combination of network and language embedding models to identify popular topics of misinformation amongst users in each language. Despite English users forming the most dominant language base on Twitter, we find that the popularity of misinformation in Japanese regularly outstrips English for certain topics. Misinformation shared by Spanish users is also over-represented in proportion to its much smaller user base. Our results provide insight into the current state of misinformation in each language. While we discuss some of the possible drivers behind the factors such as language over-representation, our study also highlights the need for further cross-lingual misinformation research in order to better understand this phenomena in a truly global context.

## Introduction

It is well established that an active, ongoing information campaign of propaganda and disinformation form a major part of Russian foreign policy^1^. This is not new in the context of Ukraine, where the impact on the information literacy of Ukrainians from such campaigns have been actively documented, particularly since the Ukrainian revolution (Euromaidan) and subsequent annexation of Crimea in 2014^2,3^. Ongoing attempts of Russian-backed media to push a narrative of propaganda and disinformation have also been observed in the case of particular events such as the downing of Malaysian Airlines Flight 17 (MH17) over Ukraine^4,5^. This campaign has arguably been taken to new levels since the invasion of Ukraine in February 2022. While western media outlets have generally condemned the invasion, this has done little to discourage Russia from continuing to actively promote propaganda and disinformation on social media in an attempt to undermine this support for Ukraine^6,7^. While Russian social media interference has been the subject of past research^5,8,9^, there remains an ongoing need to investigate this phenomena as misinformation continues to evolve in different contexts^10^.

The global nature of the Covid-19 pandemic provided a rich setting to observe misinformation in a multi-lingual setting. Such research has ranged from characterizing features of misinformation^11,12^, to misinformation resilience^13–15^ between languages and/or countries. Whilst rapidly increasing, research on misinformation in the context of the Russian invasion of Ukraine is still relatively nascent^16^. Research also tends to be in a single language, or in a limited selection of languages such as between English and Russian^10,16,17^. We therefore look to combine these two facets by applying a multi-lingual perspective to pro-Russian misinformation on social media. In this paper, we seek to identify and characterize popular topics of pro-Russian misinformation on Twitter in six different languages in the context of the opening weeks of the invasion , which saw a surge of social media activity^18^. Specifically, these languages are English, Japanese, Spanish, French, German, and Korean. We use an exploratory method combining network techniques and language embedding models to identify popular topics of misinformation on Twitter in each language, plot the retweet timing of these topics, and use URLs associated with those topics to observe cross-lingual content sharing. Regarding the use of the words “misinformation” and “disinformation”, “disinformation” is regarded as incorrect information that is created and spread with the intention of causing harm. As mentioned earlier, while the Russian state actively engages in the promotion of disinformation, this does not necessarily mean that social media users who share this information also have the intention of causing harm. We do not intend to evaluate intention of harm in this study, which in itself is a challenge. We therefore use the word “misinformation” to denote incorrect information that is shared without implying harmful intentions.

## Results

Through the use of networks and a language embedding model, we are able to identify five popular topics of pro-Russian misinformation that occurred during the first two weeks of the Russian invasion of Ukraine in our six languages. As we are looking to analyse the trends of the most popular topics across our six languages, this is not a comprehensive list of all misinformation that has occurred during the time. We then compare trends of these popular misinformation topics between the six languages. We plot the relative popularity of tweets in each language, the popularity of the misinformation topics over time, and finally a cross-lingual sharing analysis using the URLs shared by misinformation tweets in each language.

### Five topics of Misinformation

**Figure 1:**
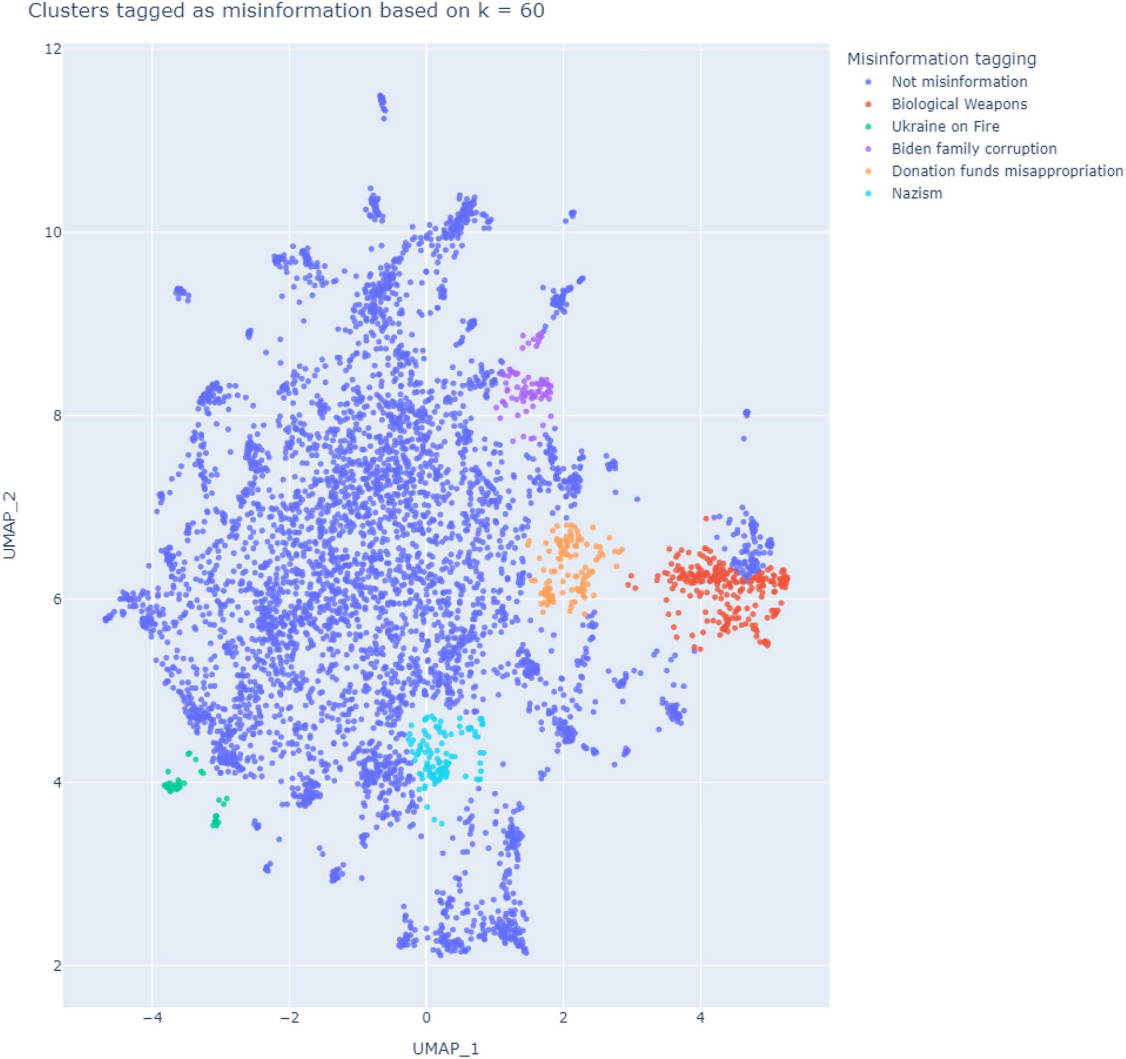
A 2-dimensional representation of popular tweets shared by pro-Russian users in English, Japanese, Spanish, French, German, and Korean. Full details on how this graph was arrived at is described in the Methods section.

Once popular highly retweeted tweets shared by Twitter users supportive of the invasion (henceforth referred to as “pro-Russian”) were identified in each language, plotting a multi-dimensional representation of these tweets revealed five clusters of explicit misinformation (Figure 1). These topics include:

1. The existence of biological weapon development facilities in Ukraine: While biological research laboratories do exist with partial funding provided by the United States, United Nation (U.N.) calls for Ukraine to temporarily close them down to prevent the potential spread of diseases have been turned into a false narrative by pro-Russian media that such facilities are producing biological weapons.
2. A popular pro-Russia documentary, “Ukraine on Fire”, which presents a misleading version of the events leading up to the invasion of Ukraine: “Ukraine on Fire” has been criticised as presenting pro-Russian perspectives on events without assessing the veracity of the claims being made, leading to a number of false narratives being perpetuated.
3. US President Joe Biden and his son Hunter using the war for personal enrichment: It is understood that Hunter Biden has had business interests in Ukraine extending to prior to the invasion, however there is no evidence to suggest that either he or Joe Biden has used the war to personally enrich themselves.
4. Misappropriation of donations to fund Hillary Clinton’s failed 2016 presidential bid: There is no evidence to suggest that the former Ukrainian president aided Hillary Clinton.
5. The presence of Nazis in Ukraine to justify the invasion: Claims of a “Neo-Nazi” regime are countered by the fact that support for far-right candidates in the last parliamentary elections was comparatively lower than in other countries.

We note that this does not necessarily mean that misinformation is non-existent in the other points plotted in Figure 1. For example, we identified a cluster of tweets accusing western media of manipulating or falsifying evidence in order to present an anti-Russian or pro-Ukraine version of events that occur during the war. Such broad claims may rely on isolated or extreme incidents as representative of the entirety of western media. Whilst western media has generally been regarded as being pro-Ukraine in their reporting, this is not indicative of widespread manipulation or false reporting^7^. These examples highlight the difficulty of classifying what is misinformation and what is not. However, we are able to link the claims for the five clusters shown in Figure 1 to specific sources that debunk those claims, which we show in Table 1.

**Table 1:** Identified misinformation cluster and source for classifying.

**Misinformation topic**	**Description**	**URL to source debunking claim**
Biden family corruption	Unproven conspiracy theory involving Joe Biden and his son Hunter’s work in Ukraine	$\bullet$ https://www.newyorker.com/news/news-desk/the-invention-of-the-conspiracy-theory-on-biden-and-ukraine
Biological Weapons	False claims that biological weapons are being developed in laboratories in Ukraine with support from the United States	$\bullet$ https://www.bbc.com/news/60711705
Donation funds misappropriation	Unproven claims of illegal Ukrainian donations to fund Clinton’s run for president	$\bullet$ https://www.statesman.com/story/news/politics/politifact /2022/02/24/fact-check-did-ukraine-donate-hillary-clintons-2016-campaign/6908839001/ $\bullet$ https://www.politifact.com/factchecks/2019/dec/04/john-kennedy2/gop-senator-falsely-says-ukraine-president-helped-/
Nazism	False claims of the overwhelming presence of neo-Nazis in Ukraine as a justification for Russia’s invasion	$\bullet$ https://www.bbc.com/news/61379405 $\bullet$ https://www.npr.org/2022/03/01/1083677765/putin-denazify-ukraine-russia-history
Ukraine on Fire	A pro-Russian propaganda documentary criticized for pushing Russian narrative falsehoods around the revolution	$\bullet$ https://www.occrp.org/en/investigations/sidebar/oliver-stone-documentary-about-kazakhstans-former-leader-nazarbayev-was-funded-by-a-nazarbayev-foundation $\bullet$ https://krytyka.com/en/articles/open-letter-oliver-stone

### Retweet proportions plots

**Figure 2:**
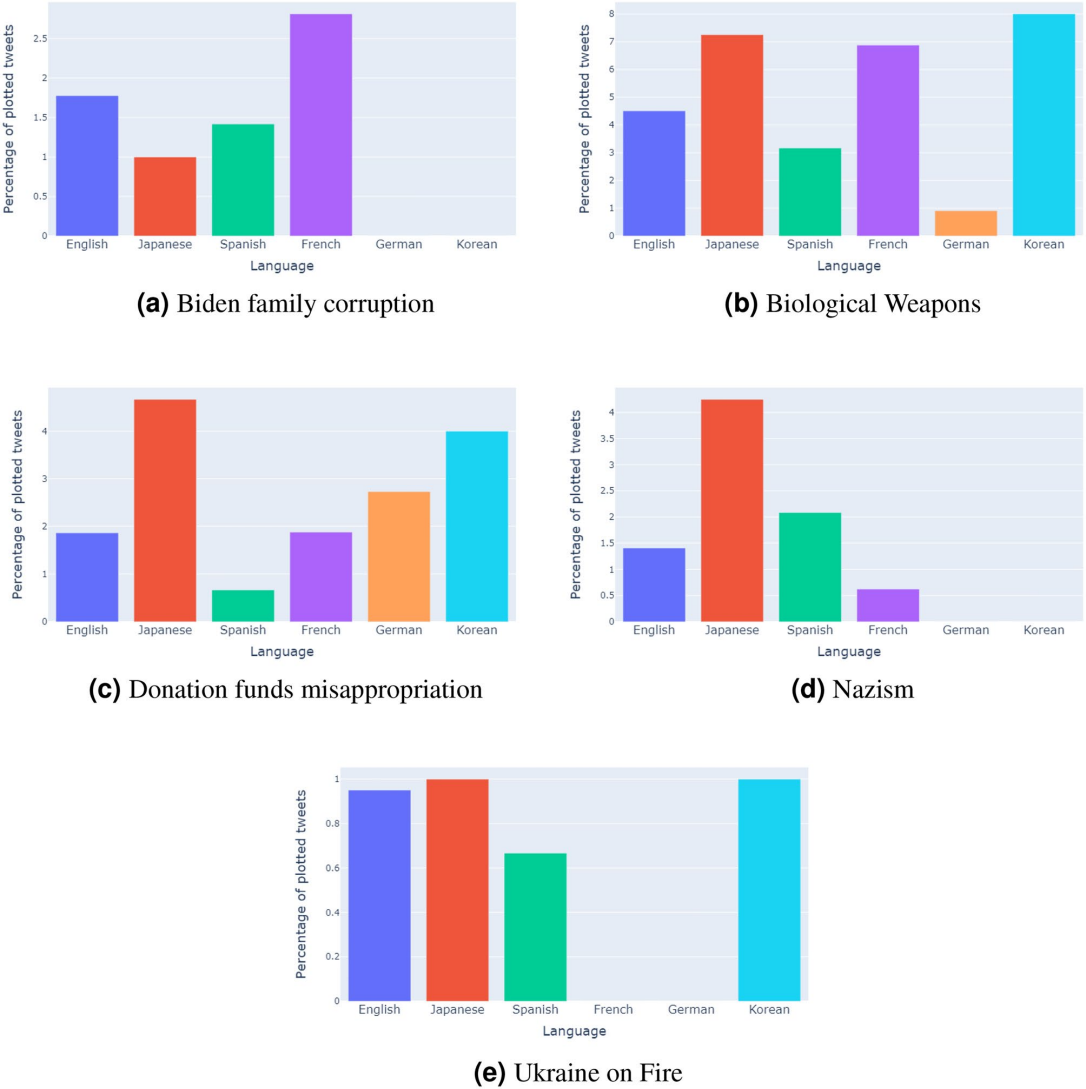
Percentage of tweets mapped in Figure 1 that pertain to each topic of misinformation by language. The use of percentages allows popular topics in minor languages to be more clearly shown.

To demonstrate the popularity of the topics of pro-Russian misinformation in each language, we plotted the number of tweets related to the five misinformation topics identified in Table 1 as a percentage of tweets in that language (Figure 2). Plotting as a percentage allows for clearer representation of popular topics in minor languages such as French, German, and Korean, and reduces the noise that niche topics may introduce. For example, English, as the largest language group in our data had over 2,400 tweets in Figure 1. In comparison, Korean only had 100 tweets. Further, English tweets are highly retweeted in comparison to Korean tweets, which is due to the much higher user base of English versus Korean speakers. Similarly, while French and German account for a small percentage of the corpus, topics such as “Biden family corruption” and “Biological weapons” are popular amongst the respective language users (Figure 2). Using this method also allows us to see in Figure 2 that a relatively high proportion of popular tweets in English and Japanese appears across all five misinformation topics. Popular Spanish tweets are also present, whilst popular French, German, and Korean tweets only appear for particular topics.

### Retweet timing plots

**Figure 3:**
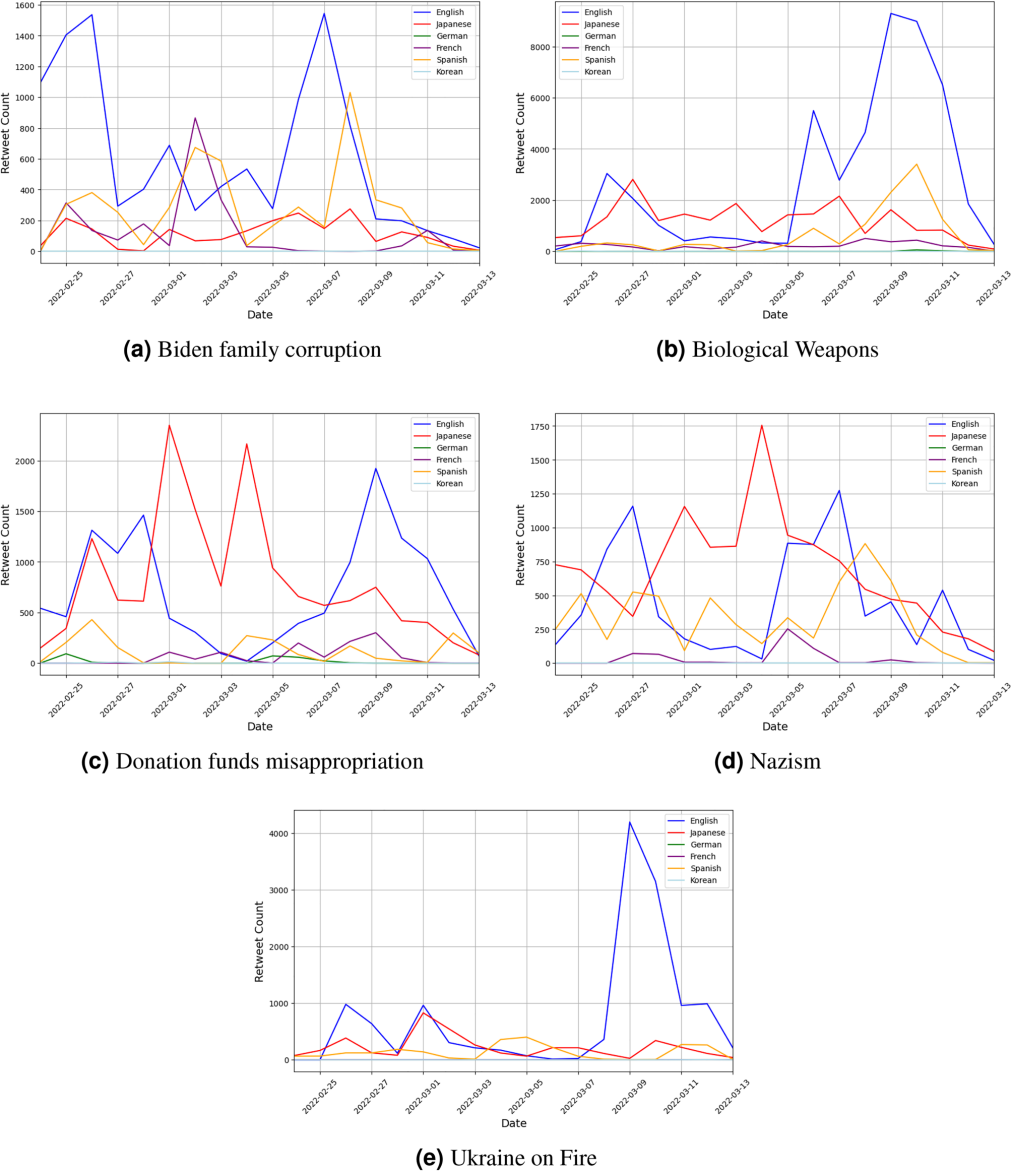
Volume of daily retweets by topic for each tweet in Figure 1.

While Figure 2 displays the overall popularity of pro-Russian topics within a given language, it is also useful to observe the timing of when particular topics became popular in certain languages. We therefore plotted the timing of when tweets related to each topic were retweeted in their respective languages in Figure 3. English tended to be the dominant language in most topics, with generally high retweet counts across all topics. However, we can also observe high levels of popularity in topics such as “Donation funds misappropriation” and “Nazism” in Japanese, which at some points are retweeted levels higher than English. A similar observation can be noted for Spanish, which is interesting considering the proportionately lower volume of Japanese and Spanish tweets compared to English.

### URL sharing

**Figure 4:**
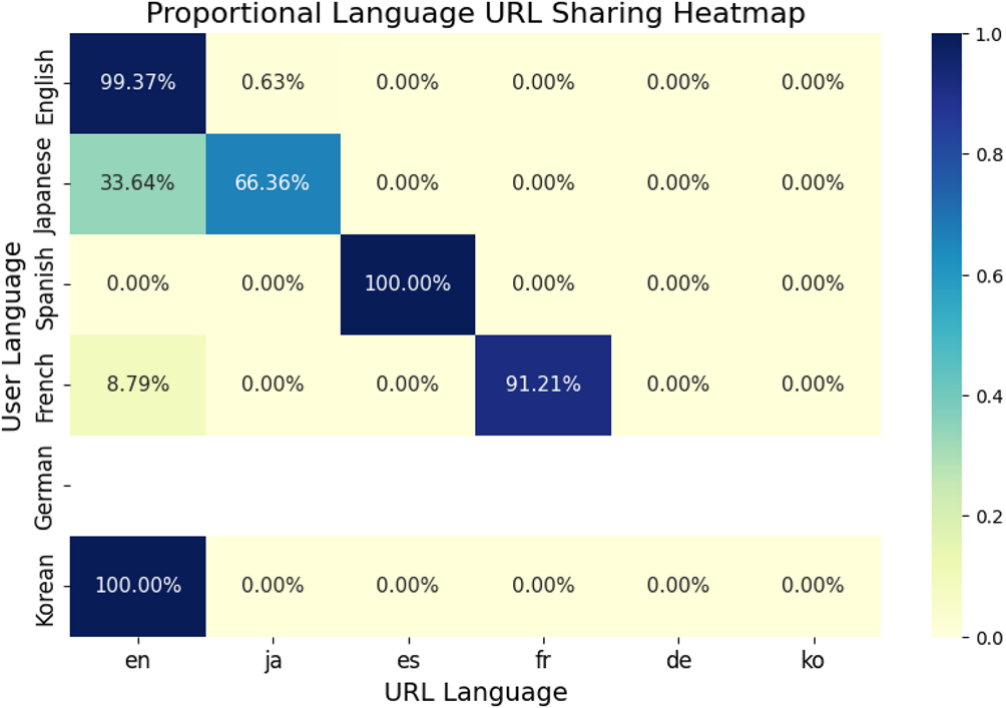
Proportion of cross-language URL sharing by tweets in each language. Blank rows indicate no URLs were shared by users in that language across 5 misinformation topics.

We then identified the language of URLs attached to the misinformation tweets plotted in Figure 1 to observe how users in one language may share misinformation in another. The results in Figure 4 show that unsurprisingly, content in the same language of the tweet being shared was the most popular. Japanese users also share a relatively large proportion of English language URLs. This represents the largest proportion of cross-lingual sharing to a non-native language. English media is known to be highly popular amongst Japanese users^19^. This phenomena, along with the relative popularity of English language URLs in French and Korean to an extent may also be explained at least in part by the “economics of misinformation”, where a combination of market size, free-speech protections in predominantly English speaking countries, and a volatile political situation contribute to the economic opportunities of creating fake news such as in the case of the Macedonian actors in the 2016 American General Elections^20^.

We note that there was no URL information for misinformation tweets for German. This is likely a result of the proportionally small tweet volume observed for German users. While these represent the most popular tweets based on retweet volume, there were no URLs attached to these most popular retweets. A similar phenomenon also occurs with Korean tweets sharing English URLs. Korean also had a similarly small tweet volume, reflecting the lower amount of content. A relatively small number number of Korean tweets had English language URLs associated with them, whilst the remainder of Korean tweets had no URLs associated with them. Hence, we consider that English URLs are likely over-represented in regards to Korean tweets.

## Discussion

The social media tactics used by the Russian state to create an environment of disinformation have been well researched, particularly in English and Russian communities, point to a well organised, coordinated community of actors^6,9,21^. Such groups include the Internet Research Agency, for which it has been suggested that there are operations in German, Italian, Arabic, French, and Spanish by^5^, although the focus of their study is the group’s U.S. operations. While we do not investigate the origins and motivations of the actors involved in our study, it is clear that the effects of Russia’s misinformation campaign regarding the invasion are truly global, and spread far beyond the English and English-Russian communities that are often the subject of research. For example,^10^ observe that misinformation regarding biological weapons laboratories has origins in a disinformation campaign started by the Russian Ministry of Defence and Russian state sponsored media. As^10^ attempt to elucidate the mechanisms at play such as notable influencers in English and Russian, they also find related content in Chinese and Arabic. The surprising popularity of the topic that we discover in Japanese, and to a lesser extent Korean that we demonstrate in Figure 2 also points to similar mechanisms at play that are worth investigating from a more detailed perspective.

As previously mentioned, the global impact of the Covid-19 pandemic also provided an opportunity for misinformation to be observed in a multi-lingual, cross-country setting which may help to explain some of the phenomena observed in this study. The United States, Mexico, and Spain have been demonstrated as being relatively susceptible to misinformation. This can be a result of individual user tendencies such as higher readiness or willingness to believe in the accuracy of a piece of misinformation, or environmental factors that exist within the country such as a polarized political environment and low-trust fragmented media environments. This has resulted in misinformation being more prevalent and easily spread. On the other hand, Germany amongst other countries demonstrated relatively low susceptibility to misinformation on account of its relatively strong media environment^13,14^. The popularity of pro-Russian misinformation amongst Spanish and English users, to which these countries contribute a large user base, suggest that these factors largely continue to exist. Understanding the prevalence of misinformation in Japanese is more challenging. It is necessary to do so however, as the Japanese community represents one of the largest on the social media network. Whilst hyper-partisan, racist, and deliberately false content still exists and is shared on Japanese social media, the country has generally been regarded as having a comparatively low prevalence of misinformation^22^. Our findings question this claim, as we find that misinformation in Japanese is relatively popular, with certain topics such as “Donation funds misappropriation” and “Nazism” reaching daily retweet peaks higher in Japanese than in English.

Identifying misinformation also continues to be a challenge in the current online environment. This is especially so in the context of this study, where a global conflict results in misinformation being shared in many different languages. Through similar use of a language embedding model,^17^ identify potential tweets containing misinformation by matching fact-checked claims against tweets in the English language. However, this method implicitly assumes that the stance of tweets identified as being similar to fact-checked claims are spreading misinformation. This may not necessarily be the case of tweets that aim to share how “the other side” presents information, or satire tweets as examples. We overcome this issue by using the retweet network to label users as pro-Russian or not prior to identifying misinformation. By taking advantage of the “dense connections” typically found in misinformation versus true information clusters demonstrated by^23^, the tweets we identify are a “truer” stance of the users that share misinformation. Our most highly retweeted topics of misinformation also demonstrated that the use of a language embedding model can identify misinformation in different languages. Characterising misinformation in studies such as the one performed by^12^ is resource intensive, often relying on researchers with linguistic expertise. Using a language embedding model can help to reduce this burden, while at the same time allowing insight into a greater variety of languages. Whilst the aim of this study was to analyse the most popular topics of misinformation in a cross-lingual context, further research will look to use these methods to more comprehensively capture misinformation in a broader context. This will allow for the discovery of more niche topics of misinformation in each language that may have been drowned out by the presence of non-misinformation topics when we observed popular tweets.

## Methods

### Data collection

**Figure 5:**
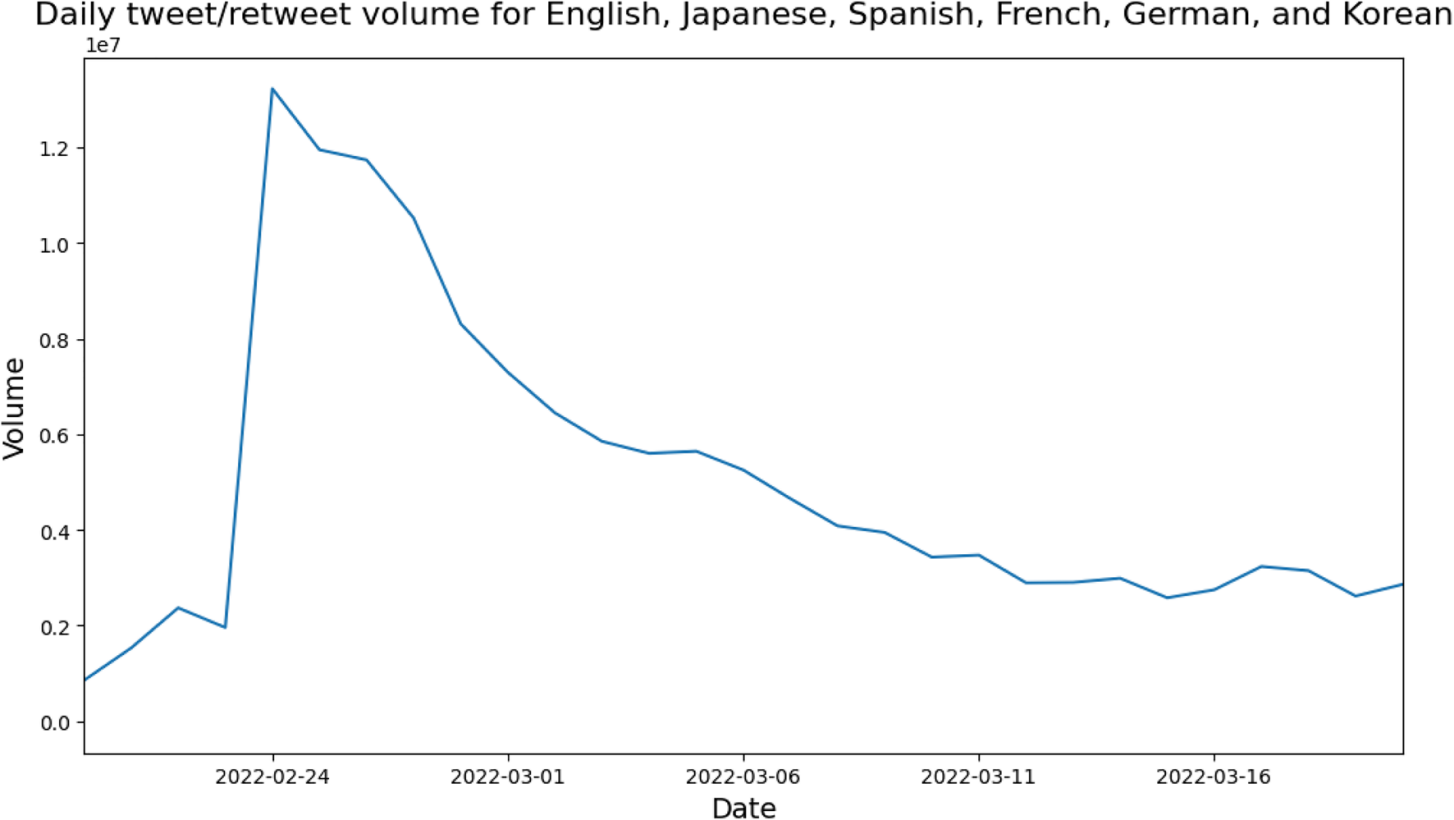
Daily volume of tweet/retweet data collected across the six languages over time.

Twitter tweets and retweets containing the 16 keywords in English and their translational equivalents in Japanese, Spanish, French, German, and Korean (Table 2) were collected using Twitter REST API v1.1. English, Spanish, French, and German were chosen as the top languages in which discourse took place according to^18^, whilst Japanese and Korean are geographically significant to the location where this study was performed. The date range for data collection was from February 24th 2022, the start of the invasion, to March 12th, when Figure 5 shows that tweet/retweet volume plateaus. This is also similar to when^18^ in their study tracking tweets related to the conflict also observe a drop in daily tweet volume from the spike in activity that resulted at the start of the invasion. Despite the relatively short time-frame, this resulted in a large data-set of approximately 53 million tweets/retweets, with 30.5 million in English, 11.8 million in Japanese, 7.5 million in Spanish, 1.7 million in French, 571K in German, and 690K in Korean. As this study used publicly available data and no human subjects were involved, it was exempt from ethical review by the Institutional Review Boards in accordance with the authors’ institutional guidelines. Data was also further anonymized through aggregation.

**Table 2:**
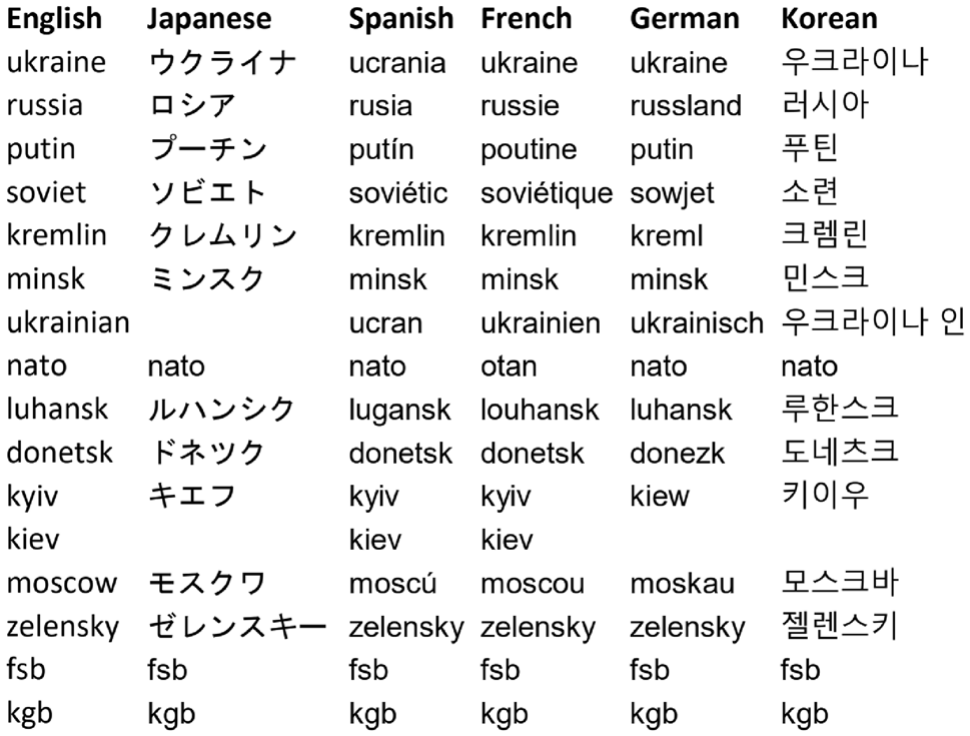
Keywords used to filter retweet data in each language. Note that certain keywords for languages other than English may be already covered by a previous version, hence the blank spaces (e.g. Kiev in Japanese is already considered under Kyiv, as there is only one equivalent translation of the name of the Ukrainian capital in Japanese).

### Influential user network tagging

In order to identify retweets containing pro-Russian content, we first looked for users that have a pro-Russia stance. Such individuals are naturally the most likely to share Russian propaganda and/or misinformation in their tweets/retweets. This also negates the limitations in^17^ of stance identification, as we can assume that users who are pro-Russia are likely to be supportive of the piece of pro-Russian misinformation being shared. To identify these users, we first assume that users who retweet more pro-Russia users than non pro-Russia users are likely to be pro-Russia themselves. Based on this, we rebuild the retweet network and approximate the pro-Russia stance of the user based on their retweet network in a multi-step process.

**Figure 6:**
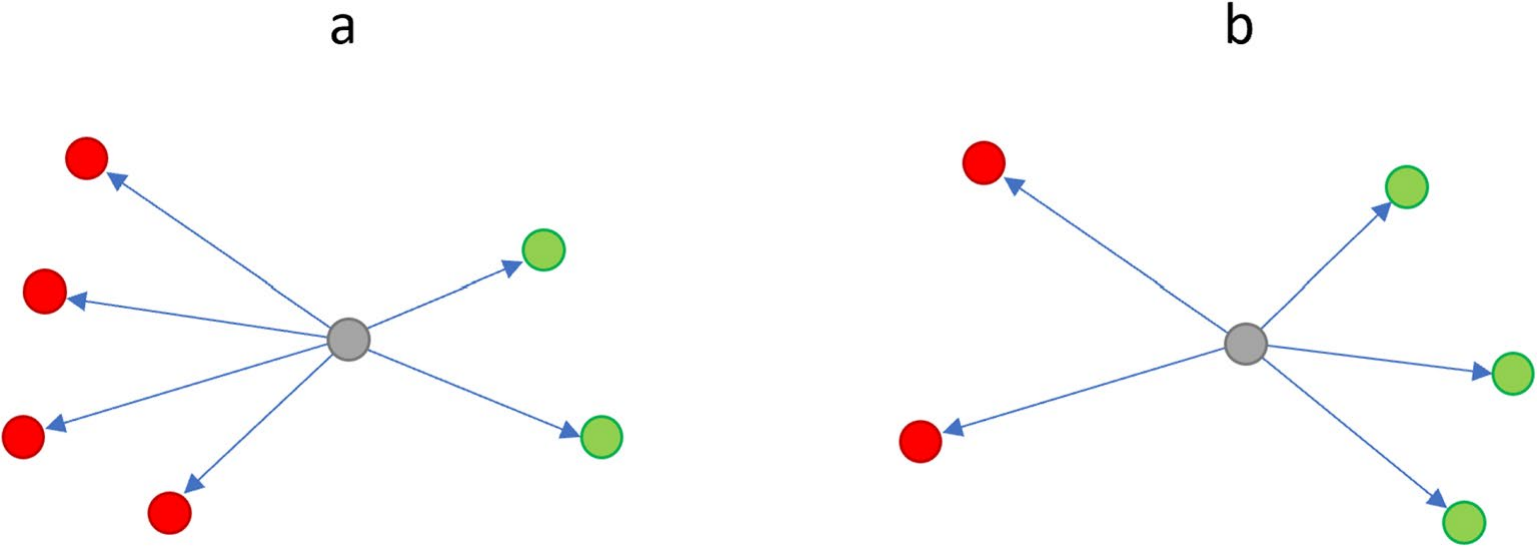
User tagging example: If an un-tagged user (grey) retweets users known to share misinformation (red) more times than those that do not (green), the user is considered a misinformation spreader. If a user retweets more non-misinformation spreaders (green), then they are not considered misinformation spreaders. The former situation is shown in a, the latter is shown in b.

- *Step One - Build the overall retweet network:* We built the retweet network based on all tweets/retweets collected. Using this network, we calculated the node degrees for each individual as a measure of their influentiality within the network.
- *Step Two - Manually identify users who retweet misinformation and users that do not:* We identified users who shared URLs to websites known for sharing Russian “misinformation and propaganda URLs” such as RT, Tass, Sputnik, and Topwar^1^. Sites such as RT and Sputnik continue to play a role in Russia’s disinformation campaign (https://www.state.gov/report-rt-and-sputniks-role-in-russias-disinformation-and-propaganda-ecosystem/). At least RT and Sputnik are known to also publish content in the other languages observed in this study in addition to English, although content in Korean is no longer available at the time of this writing. Ordering such users based on their node degrees calculated in Step One, we manually reviewed the most influential users who also shared the most Russian propaganda websites to identify genuine propaganda/misinformation spreaders and tagged them as “misinformation spreaders”. The manual review allowed us to remove users who shared propaganda URLs, but are not genuinely trying to spread it in an attempt to convince others. As described earlier, such examples can occur in the case of users demonstrating how the “other side” presents information without actually believing in it themselves, or satire accounts. Once at least 20 users were manually tagged as “propaganda spreaders”, a similar number of users were identified, manually reviewed, and tagged as “non-propaganda spreaders”.
- *Step Three- Use manually tagged users to approximate remaining users in the network:* The final step is to then tag the remaining users connected in the network to the manually tagged users from Step 2 based on their interactions. If an un-tagged user retweets a user who has been tagged as sharing propaganda more times than a user who has been tagged as non-propaganda, then that user would be tagged as a propaganda sharer, and vice versa (Figure 6). This process was repeated to 6 node hops for Japanese, Spanish, French, German, and Korean, whilst the process was done to 3 node hops for English due to computational limitations owing to the size of the English data set. Despite the fewer node hops for English, the number of users tagged as either “propaganda spreaders” or “non-propaganda spreaders” was consistently between 60 to 70% of the population across all languages, including English. The risk of “drift” (i.e. where users are incorrectly tagged as a propaganda spreader and vice versa) is also considered negligible due to the combination of aforementioned “dense connection” clusters phenomena found in^23^, and the fact that the most influential users were tagged first. These factors are expected to lessen the impact of any possible less influential, distant mis-tagged users may have.

After building the network and identifying pro-Russian users, we find that this results in a total of approximately 470K (out of a total of 9.2 million) individuals being identified as pro-Russian. Approximately 220K users were identified in English (representing 6% of all tagged English users), 40K in Japanese (5% of Japanese users), 177K in Spanish (20% of Spanish users), 26K in French (10% of French users), 7K in German (7% of German users), and 120 in Korean (<1% of Korean users). Whilst it is difficult to quantify the actual number of people who support Russia, our network rebuilding exercise suggests that such users are the minority across all languages. The somewhat high prevalence of pro-Russia users in Spanish may also be explained by the fact that Mexico and Spain, the two countries that contribute to the largest Spanish speaking population in the world is considered to be relatively susceptible to misinformation, as demonstrated during the Covid-19 pandemic^13^. However, more research is required into understanding the cultural factors at play amongst Spanish users.

Against this background, we explored the most popular pro-Russian tweets/retweets in order to uncover key topics of misinformation that were popular in each language. We perform a cross-lingual content comparison analysis using text embedding , and then performing k-means clustering on the embeddings to identify topics/themes of misinformation in a method similar to that used by^25^. Based on the identified themes, we then compared the popularity of these themes between the different languages, and the timing of when particular themes were retweeted. Finally, we used the language of URLs shared to observe how users shared information in other languages.

### Identifying misinformation

The aim of this research is to explore popular themes of pro-Russian misinformation and their alignment. Highly retweeted tweets were used in this case, as it is reasonable to suggest that tweets that are widely retweeted express a popular idea or sentiment. Further, we wanted to translate tweets in order to confirm their content from different languages, and a widely retweeted tweet has the advantage of only needing to be translated once as opposed to translating a wide variety of tweets. We therefore sampled the top 1 percent of retweeted tweets based on overall retweet counts, with a floor of 100 to include representation from languages with smaller volume such as Korean. We find that this top 1 percent represents on average 33% of possible misinformation retweet volume, justifying this decision (Table 3).

**Table 3:** Top one percent retweeted tweets and proportion of overall retweet population that they represent.

**Language**	**No. of top 1% tweets**	**Top 1% tweet retweet count / population of retweets**	**Proportion**
English	2420	708,783 out of 1,645,338	43%
Japanese	1200	286,449 out of 809,928	35%
Spanish	1200	345,220 out of 998,796	35%
French	320	70,619 out of 202,907	35%
German	110	8,615 out of 40,804	21%
Korean	100	344 out of 1,055	33%

**Figure 7:**
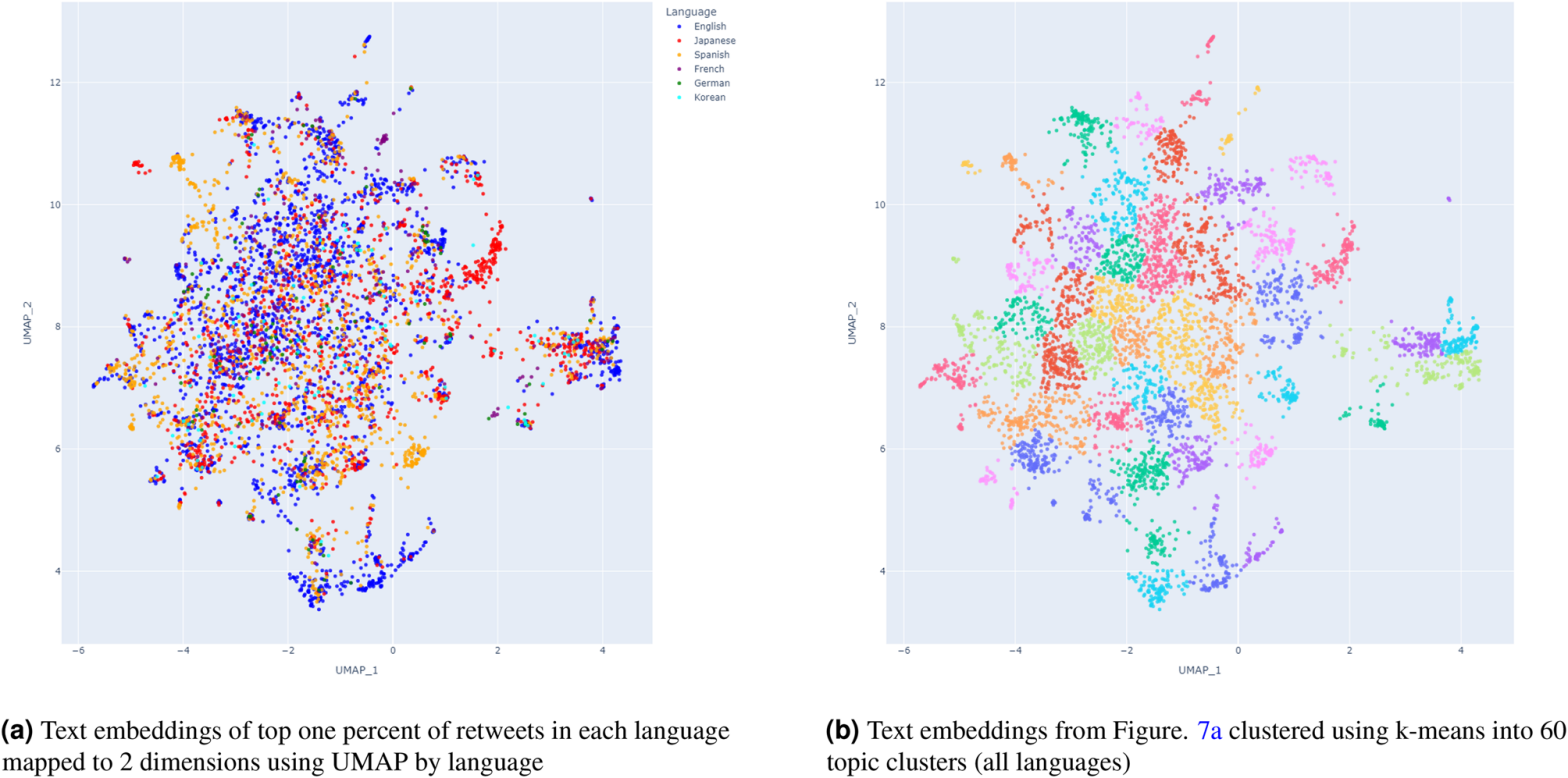
Text embeddings of top one percent of retweets in each language mapped using UMAP (Subfig. 7a). The same embeddings were then clustered using k-means clustering to identify topic clusters (Subfig. 7b). The topic clusters in Subfig. 7b directly correspond to the 5 labelled clusters in Figure 1.

We translated the top 1 percent of retweets from each language to English using the Google Translate API. We then derived text embeddings from these tweets by applying all-MiniLM-L6-v2, and mapped them to 2-dimensional space with UMAP (Figure 7 (left)). all-MiniLM-L6-v2 is a popular Bidirectional Encoder Representations from Transformers (BERT) model that generates contextual sentence vector representations (https://huggingface.co/sentence-transformers/all-MiniLM-L6-v2). Each tweet is represented in a 384 dimensional vector space, and represents the topic of the tweet content^26^. UMAP is also a widely used method of reducing the number of dimensions whilst still preserving local and global features, allowing each tweet represented in 384 dimension form to be plotted into the 2 dimensional form shown in Figure 7 (https://github.com/lmcinnes/umap).^25^ Finally, we used k-means clustering to determine groups of themes/topics, and to identify which language clusters discussed certain themes/topics the most (Figure 7 (right)). K-means clustering is a popular method for clustering analysis, where the distance between the cluster points and their assigned mean is minimized, based on a predetermined number of clusters.

**Figure 8:**
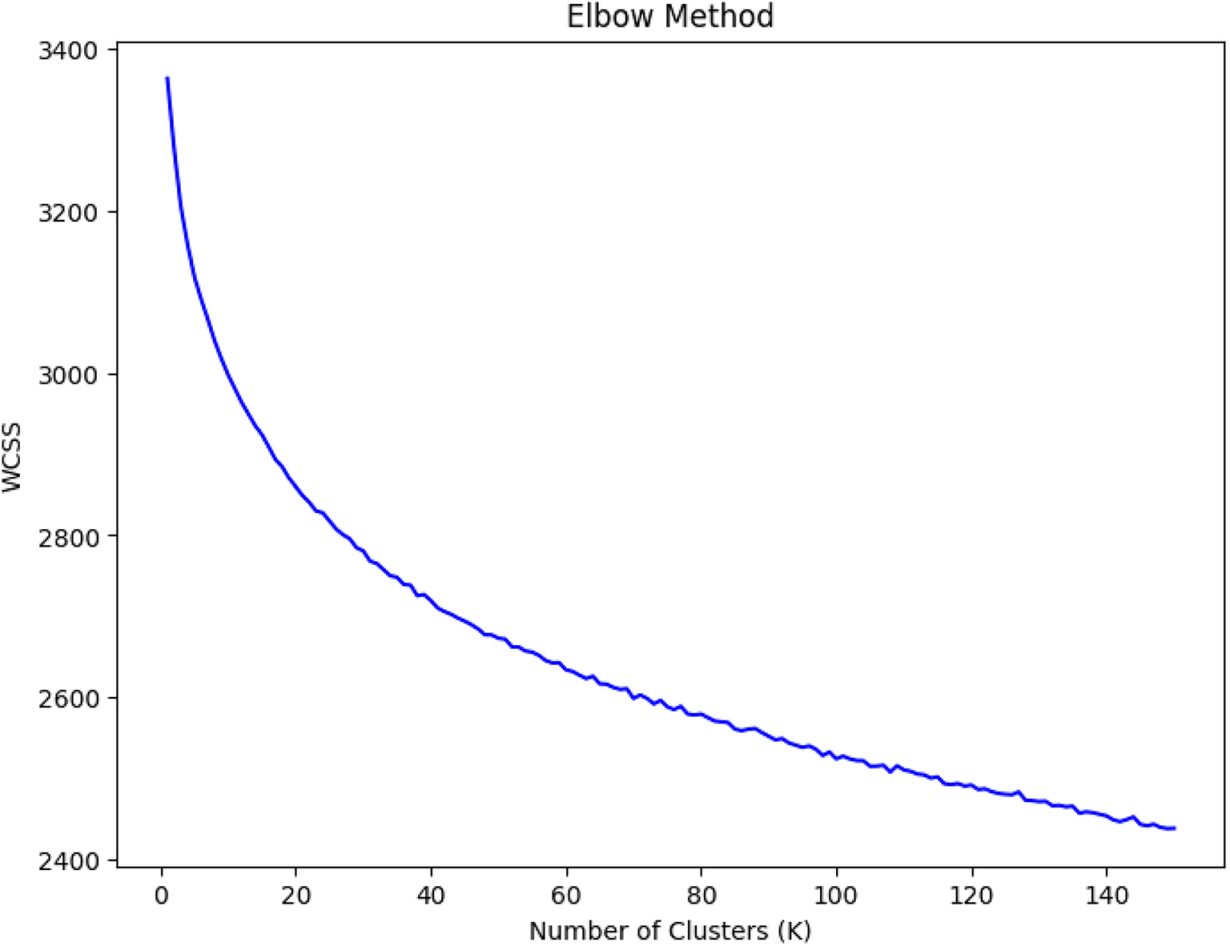
Elbow method to determine the optimal value of k topic clusters in Figure 7b.

To find the optimal number of topic clusters shown in Figure 7b, we used the elbow method. For a given number of clusters, the sum of squared distance between each point of the cluster and the centre of the cluster is measured. This process is repeated within a range of clusters until the distance is reduced enough that it is not practicable to increase the cluster count. The optimal point is where there is a minimal reduction in the distance for the increase in cluster size. Figure 8 suggests that this optimal point is at a cluster count of around 60.

A manual review of the tweets in each cluster formed through k-means in Figure 7a suggested that the majority of clusters are not indicative of misinformation, although this does not necessarily mean that they contain no misinformation. Examples of general topics not explicitly tied to pro-Russian propaganda or misinformation include tweet clusters concerned about the relationship between Japan and Russia, or domestic discussions about how world leaders such as French President Emmanuel Macron or Mexican President Andrés Manuel López Obrador handle the situation.

As mentioned earlier, even where a cluster is tagged as not sharing explicit misinformation, this does not necessarily mean that absolutely no misinformation was shared within that cluster, either within specific tweets or in general. In fact, we find heavy suggestions of misinformation being shared. For example, a tweet cluster that suggests that western media manipulate or falsify evidence in order to present an anti-Kremlin or pro-Ukraine version of events that occur during the war. Such broad claims often rely on what may be isolated or extreme incidents as representative of the entirety of the industry. Whilst western media has generally been pro-Ukraine in their reporting^6^, this is not indicative of widespread manipulation or false reporting. Another example is fears of NATO expansionism being used as justification for the invasion. Such claims have a complex history behind it that makes determining what is explicitly true or false difficult^27^. These examples demonstrate the challenges of identifying and classifying misinformation in research. For these reasons, we only categorised a topic cluster as misinformation if we are able to link the topic to a fact-checking source that debunks the claims being made. Whilst this may seem to be an overly narrow definition of what constitutes misinformation, we argue that being too broad also poses its own challenges and risks. We therefore identified 5 topic clusters where the content of the tweets in the respective cluster shared explicit misinformation. These 5 topics are shown in Figure 1, and we are able to link the claims being made to sources that specifically debunk the claims being made in the tweet topic cluster, which we demonstrate in Table 1.

## Data Availability

The datasets used in this study are available from the corresponding author on reasonable request. Most can be retrieved from twitter.com, except for deleted tweets. A list of the tweet ids used in this study is also available as supplementary material.
